# Contrasting α- and β-Diversity Responses of Aboveground Vegetation and Soil Seed Banks to Experimental Warming over Two Consecutive Growing Seasons

**DOI:** 10.3390/plants15162435

**Published:** 2026-08-11

**Authors:** Yuning Zhang, Shanheng Shi, Xuankai Zhuang, Ziyan Zhou, Xiaoni Liu, Yafei Shi

**Affiliations:** 1 Pratacultural College, Gansu Agricultural University, Lanzhou 730070, China; 1073324020042@st.gsau.edu.cn (Y.Z.); 1073325020088@st.gsau.edu.cn (X.Z.); 1073325120202@st.gsau.edu.cn (Z.Z.); liuxn@gsau.edu.cn (X.L.); 2 College of Agronomy and Biotechnology, China Agricultural University, Beijing 100193, China; ss19953215914@163.com

**Keywords:** alpine meadow, open-top chamber (OTC), species turnover, community assembly, climate change

## Abstract

Climate warming may alter both α and β diversity in alpine meadows, yet whether aboveground vegetation and soil seed banks respond synchronously remains unclear. Using a two-year open-top chamber (OTC) warming experiment, we investigated the responses of community composition, abundance, alpha diversity, and beta diversity in aboveground vegetation and the soil seed bank under experimental warming. Experimental warming significantly increased aboveground biomass to 1.73 times that of the CK treatment under the high-OTC treatment, while reducing species richness and the Shannon diversity index by 29.07% and 22.64%, respectively. Warming also promoted species turnover, increasing the beta diversity of aboveground vegetation by 24% and 37% under the low- and high-OTC treatments, respectively. In contrast, the soil seed bank mainly exhibited an increase in seed density, reaching 2.47 times that of the CK treatment under the high-OTC treatment, whereas species richness increased only slightly, and beta diversity remained relatively stable despite a significant, but comparatively smaller, shift in community composition. Overall, aboveground vegetation exhibited stronger responses to short-term warming, whereas the soil seed bank showed relatively stable diversity and community characteristics under the same warming treatments. These contrasting response patterns suggest that the greater stability of the soil seed bank may provide an important source of propagules for future vegetation regeneration and community restoration. Our findings reveal contrasting aboveground-belowground responses to warming and provide new insights into predicting the responses of alpine meadow plant communities to future climate warming.

## 1. Introduction

Global climate warming is one of the major drivers of biodiversity change in terrestrial ecosystems [1,2], particularly in alpine ecosystems that are highly sensitive to increasing temperatures. Alpine meadows are characterized by long-term low-temperature limitation, short growing seasons, low productivity, and slow ecological processes, and are therefore regarded as one of the ecosystems most vulnerable to climate warming [3,4]. As global temperatures continue to rise, the diversity of aboveground vegetation and soil seed banks in alpine meadows is expected to change accordingly [5,6]. However, because these two components differ in their ecological functions and regeneration processes, their responses to climate warming may not be consistent [7]. Therefore, a comprehensive understanding of the responses of alpine meadows to climate warming requires simultaneous consideration of both aboveground vegetation and soil seed banks.

Aboveground vegetation is the most direct component of terrestrial ecosystems responding to climate warming and has therefore become the primary focus of experimental warming studies [8,9]. Based on a four-year warming experiment conducted in an alpine meadow, Zhou et al. [10] systematically monitored leaf traits, photosynthetic characteristics, and other plant functional traits, demonstrating that warming can influence plant growth and community structure by altering resource acquisition, regulating photosynthetic capacity, and modifying interspecific interactions. A global meta-analysis conducted by Lin et al. [11] further showed that warming generally enhances ecosystem primary productivity and aboveground biomass. Changes in plant diversity under warming, however, have been reported to vary among ecosystems and environmental conditions [12]. Because aboveground vegetation is the primary source of propagules for the soil seed bank, its response to warming may further influence the soil seed bank through changes in seed production and seed input processes [13]. Therefore, understanding the responses of the soil seed bank requires consideration of its ecological linkage with aboveground vegetation, providing a foundation for examining belowground regeneration processes under climate warming.

As the potential vegetation community, the soil seed bank serves as an important reservoir of propagules for future vegetation regeneration, community restoration, and ecosystem maintenance, while also acting as a bridge linking historical vegetation with future community succession [5,14]. In recent years, although several studies have investigated the effects of climate change on soil seed banks, research in this area remains limited [15]. For example, using an infrared warming experiment in alpine grasslands, Hoyle et al. [16] demonstrated that temperature is a key factor regulating seed germination and dormancy release, and that increased temperature can enhance the species richness of soil seed banks. Similarly, Ma et al. [17] found, based on soil seed banks along an elevational gradient in alpine grasslands, that temperature is one of the key drivers of soil seed bank dynamics, with higher temperatures leading to increases in both species richness and seed density. Therefore, simultaneously examining the responses of aboveground vegetation and soil seed banks is essential for understanding the adaptive processes and potential regeneration mechanisms of aboveground and belowground community diversity under climate warming [18].

The responses of alpha diversity and beta diversity to climate warming may differ between aboveground vegetation and the soil seed bank because they represent different stages of the plant life cycle and operate over different temporal scales [19,20]. Studies by Elmendorf et al. and Alexander et al. [21,22] have shown that aboveground vegetation responds directly to warming through changes in plant growth, interspecific competition, and species turnover, which may reduce local species richness while simultaneously promoting community differentiation. In contrast, Baskin and Baskin and Fenner and Thompson [23,24] proposed that the soil seed bank integrates long-term processes of seed production, dispersal, dormancy, and persistence, thereby buffering changes in the microenvironment and maintaining relatively high community stability. Although aboveground vegetation and the soil seed bank are theoretically expected to exhibit distinct response mechanisms, previous studies have mainly focused on the responses of alpha diversity within individual communities under long-term warming [25,26]. Comparatively little attention has been paid to responses during the early stages of warming or to the contrasting responses between aboveground vegetation and the soil seed bank, particularly from the integrated perspectives of both alpha diversity and beta diversity. This knowledge gap limits our understanding of the mechanisms underlying aboveground–belowground plant community responses to climate warming in alpine meadows.

To address these knowledge gaps, we conducted a two-year open-top chamber (OTC) warming experiment in an alpine meadow on the northeastern Qinghai–Tibetan Plateau and simultaneously investigated the diversity of aboveground vegetation and soil seed banks. The objectives of this study were to determine how experimental warming affects the alpha diversity, beta diversity, and community composition of aboveground vegetation and the soil seed bank, and to compare the responses of these two community components to short-term warming. Based on the contrasting ecological characteristics of aboveground vegetation and the soil seed bank, we proposed the following hypotheses: (1) experimental warming would decrease plot-level alpha diversity and increase beta diversity in aboveground vegetation because of enhanced species turnover; (2) the soil seed bank would exhibit relatively small changes in alpha diversity, beta diversity, and community composition because of its buffering capacity and legacy effects; and (3) aboveground vegetation would exhibit stronger responses to experimental warming than the soil seed bank. These hypotheses were evaluated using plot-level alpha diversity indices (species richness, Shannon, Gini–Simpson, Pielou, and Margalef indices), beta diversity, and community composition analyses. This study provides new insights into the adaptive mechanisms of alpine meadow plant communities under climate warming.

## 2. Results

### 2.1. Warming Performance During the Monitoring Period

**Figure 1:**
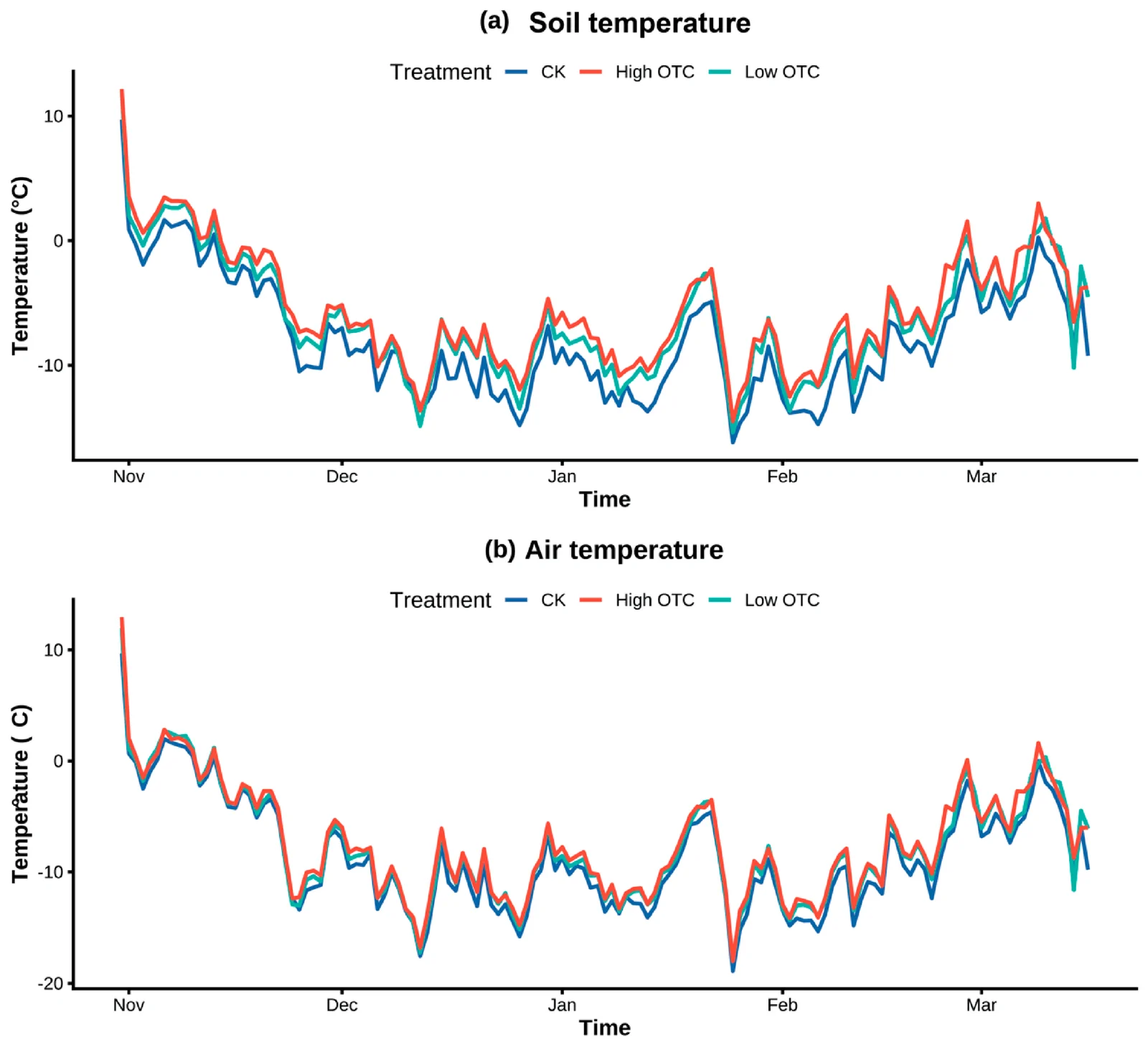
Changes in soil temperature (**a**) and air temperature (**b**) under different warming treatments. Note: (**a**) Soil temperature measured at 2 cm below the soil surface; (**b**) air temperature measured at 20 cm above the ground surface. High OTC and Low OTC represent the high- and low-intensity warming treatments, respectively, and CK represents the control shared by both warming treatments.

To evaluate the warming performance of the OTC treatments, soil surface temperature (Figure 1a) and air temperature (Figure 1b) were monitored during the available monitoring period (November 2024 to March 2025). Compared with the CK treatment, the high OTC treatment increased mean soil and air temperatures by 2.38 °C and 1.13 °C, respectively, whereas the low OTC treatment increased them by 1.66 °C and 0.86 °C, respectively. During the monitoring period, the warming effect of the high OTC treatment was consistently greater than that of the low OTC treatment. These results indicate that the two OTC configurations generated distinguishable warming conditions during the monitored period. Because continuous microclimate records covering the entire experimental period were unavailable, these temperature measurements are presented to characterize the warming performance of the OTC treatments during the monitoring period rather than throughout the entire experiment.

### 2.2. Effects of Experimental Warming on Aboveground Vegetation and Soil Seed Banks

**Figure 2:**
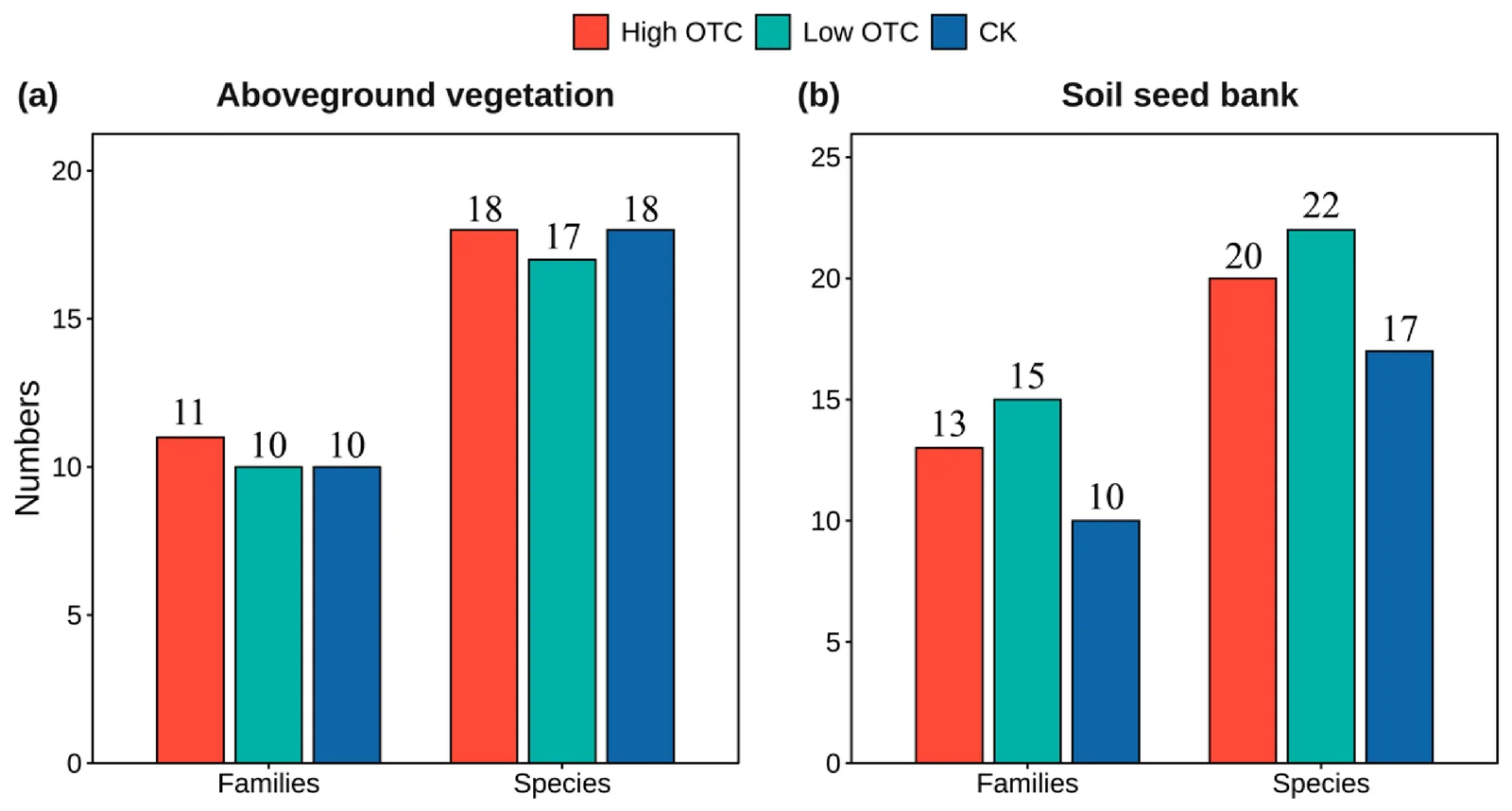
Numbers of families and species in aboveground vegetation (**a**) and the soil seed bank (**b**) across warming treatments. Note: (**a**) Species richness of the aboveground vegetation; (**b**) species richness of the soil seed bank. High OTC and Low OTC represent the high- and low-intensity warming treatments, respectively, and CK represents the control shared by both warming treatments.

The survey showed that the aboveground vegetation was dominated by species from the *Poaceae* and *Cyperaceae* families, whereas the soil seed bank was primarily dominated by species from the *Poaceae* family (Supplementary Tables S1 and S2; Figure 2a,b). Figure 2 summarizes the pooled species richness (gamma diversity) and taxonomic composition across all plots within each treatment. A total of 21 plant species belonging to 12 families were recorded in the aboveground vegetation, including 18, 17, and 18 species in the high OTC, low OTC, and CK treatments, respectively (Figure 2a). In the soil seed bank, a total of 29 plant species belonging to 19 families were identified, with 20, 22, and 17 species recorded in the high OTC, low OTC, and CK treatments, respectively (Figure 2b).

**Figure 3:**
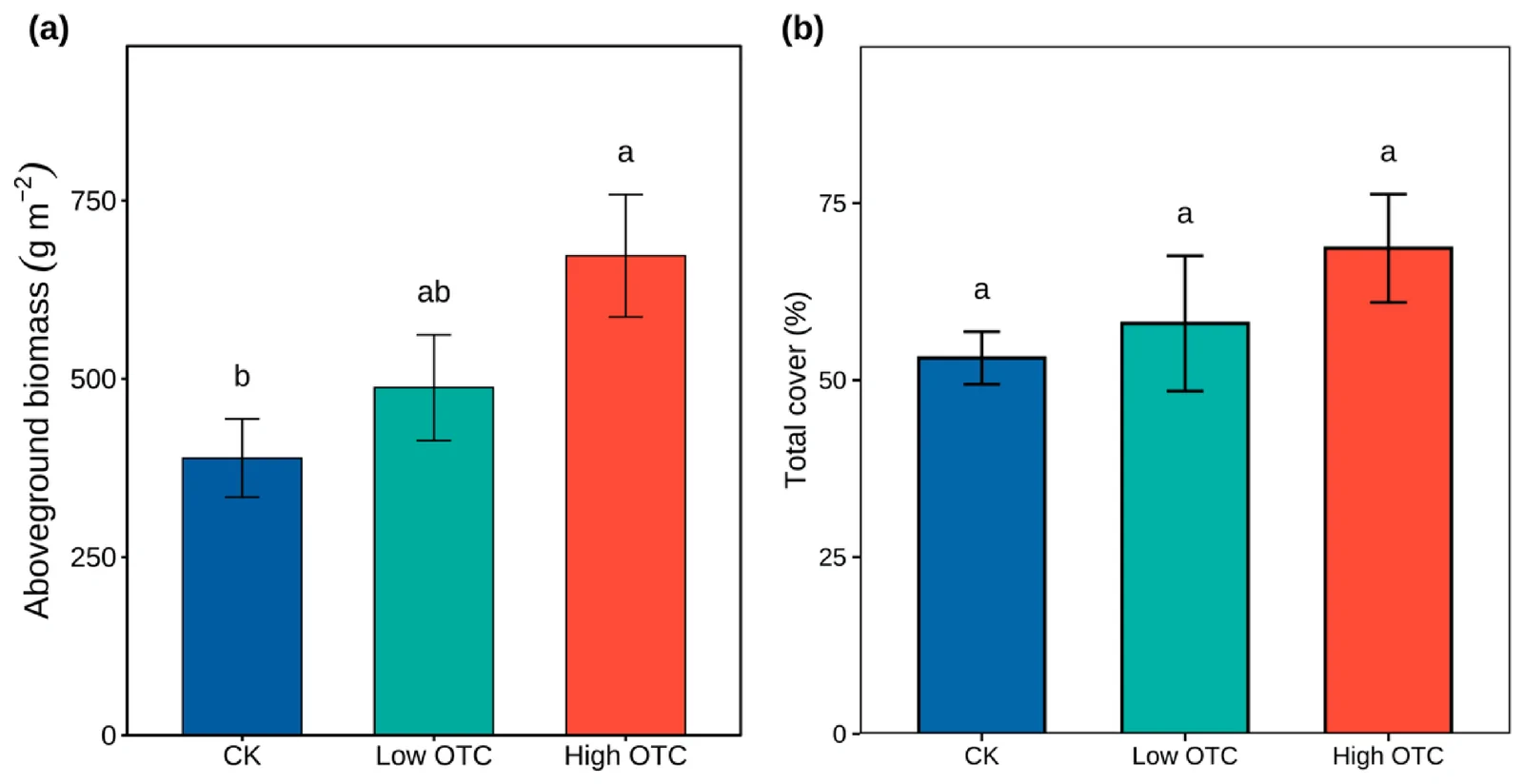
Effects of short-term experimental warming on aboveground biomass (**a**) and total vegetation cover (**b**). Note: (**a**) Aboveground biomass; (**b**) total vegetation cover. High OTC and Low OTC represent the high- and low-intensity warming treatments, respectively, and CK represents the control shared by both warming treatments. Bars represent the mean ± standard error (SE) of eight independent plots per treatment. Different lowercase letters indicate significant differences among treatments based on one-way ANOVA followed by Tukey’s honestly significant difference (HSD) test (*p* < 0.05), whereas the same lowercase letters indicate no significant difference.

Aboveground biomass (Figure 3a) and total vegetation cover (Figure 3b) showed positive responses to short-term experimental warming. Compared with the CK treatment, aboveground biomass significantly increased by 1.73-fold under the high OTC treatment (*p* < 0.05), while the increase under the low OTC treatment (1.25-fold) was not statistically significant (*p* > 0.05). Total vegetation cover increased by 9.2% and 29.2% under the low OTC and high OTC treatments, respectively, relative to the CK treatment. Moreover, the promotive effect of the high OTC treatment on vegetation cover was greater than that of the low OTC treatment.

**Figure 4:**
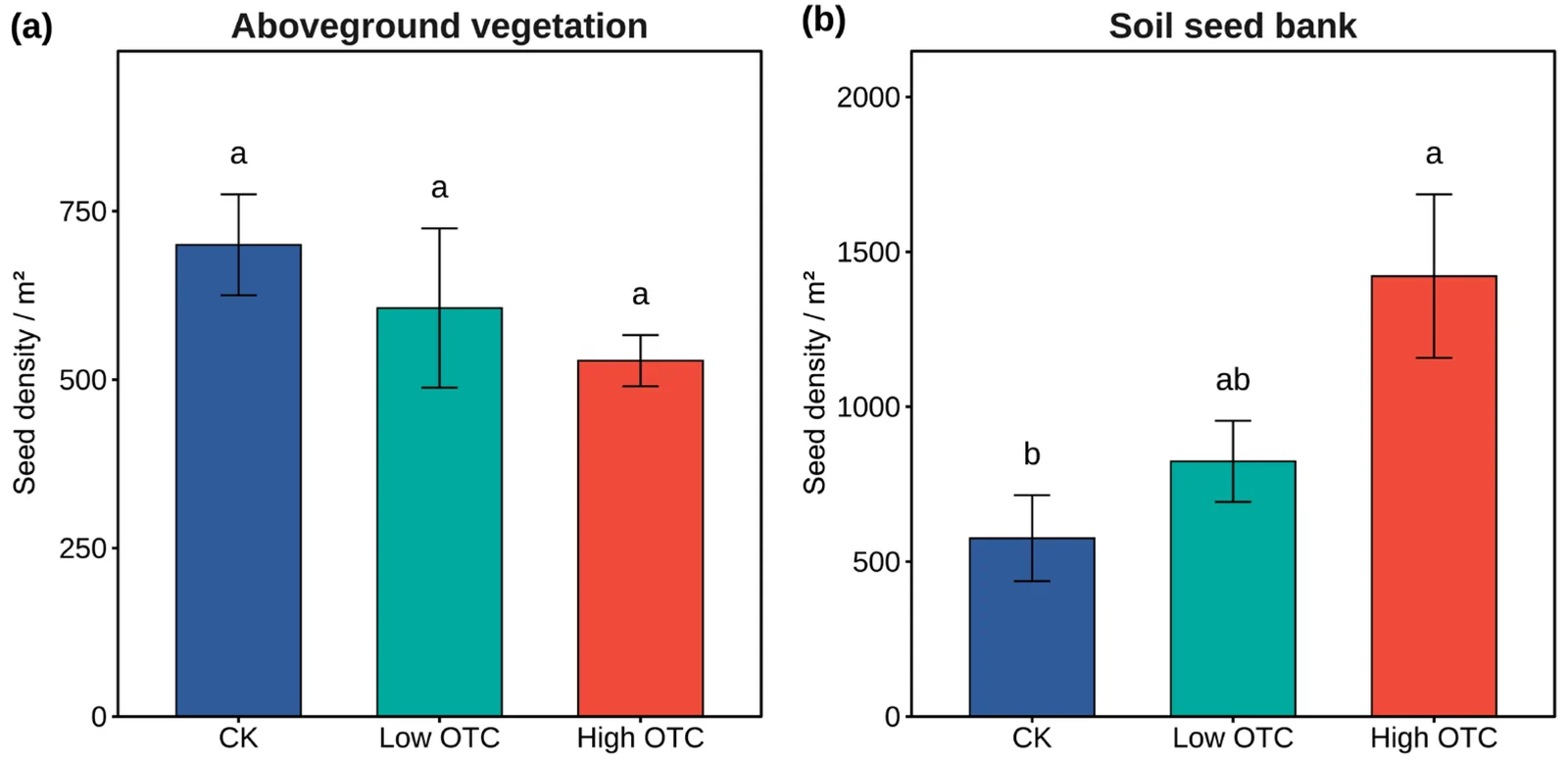
Effects of short-term experimental warming on the density of aboveground vegetation (**a**) and the soil seed bank (**b**). Note: (**a**) Aboveground vegetation; (**b**) soil seed bank. High OTC and Low OTC represent the high- and low-intensity warming treatments, respectively, and CK represents the control shared by both warming treatments. Data are presented as the mean ± standard error (SE) (n = 8 independent plots per treatment). Different lowercase letters indicate significant differences among treatments based on one-way ANOVA followed by Tukey’s honestly significant difference (HSD) test (*p* < 0.05), whereas the same lowercase letters indicate no significant difference.

Aboveground vegetation density (Figure 4a) and soil seed bank density (Figure 4b) exhibited contrasting responses to two years of experimental warming. With increasing warming intensity, aboveground vegetation density showed a declining trend, whereas soil seed bank density increased progressively. Compared with the CK treatment, the low OTC and high OTC treatments reduced aboveground vegetation density by 13.40% and 24.60%, respectively, with the effect being marginally significant (*p* = 0.06). In contrast, soil seed bank density increased with increasing warming intensity, reaching 1.43 and 2.47 times that of the CK treatment under the low OTC and high OTC treatments, respectively. The increase was statistically significant under the high OTC treatment (*p* < 0.05).

### 2.3. Effects of Warming on Alpha Diversity of Aboveground Vegetation and the Soil Seed Bank

**Figure 5:**
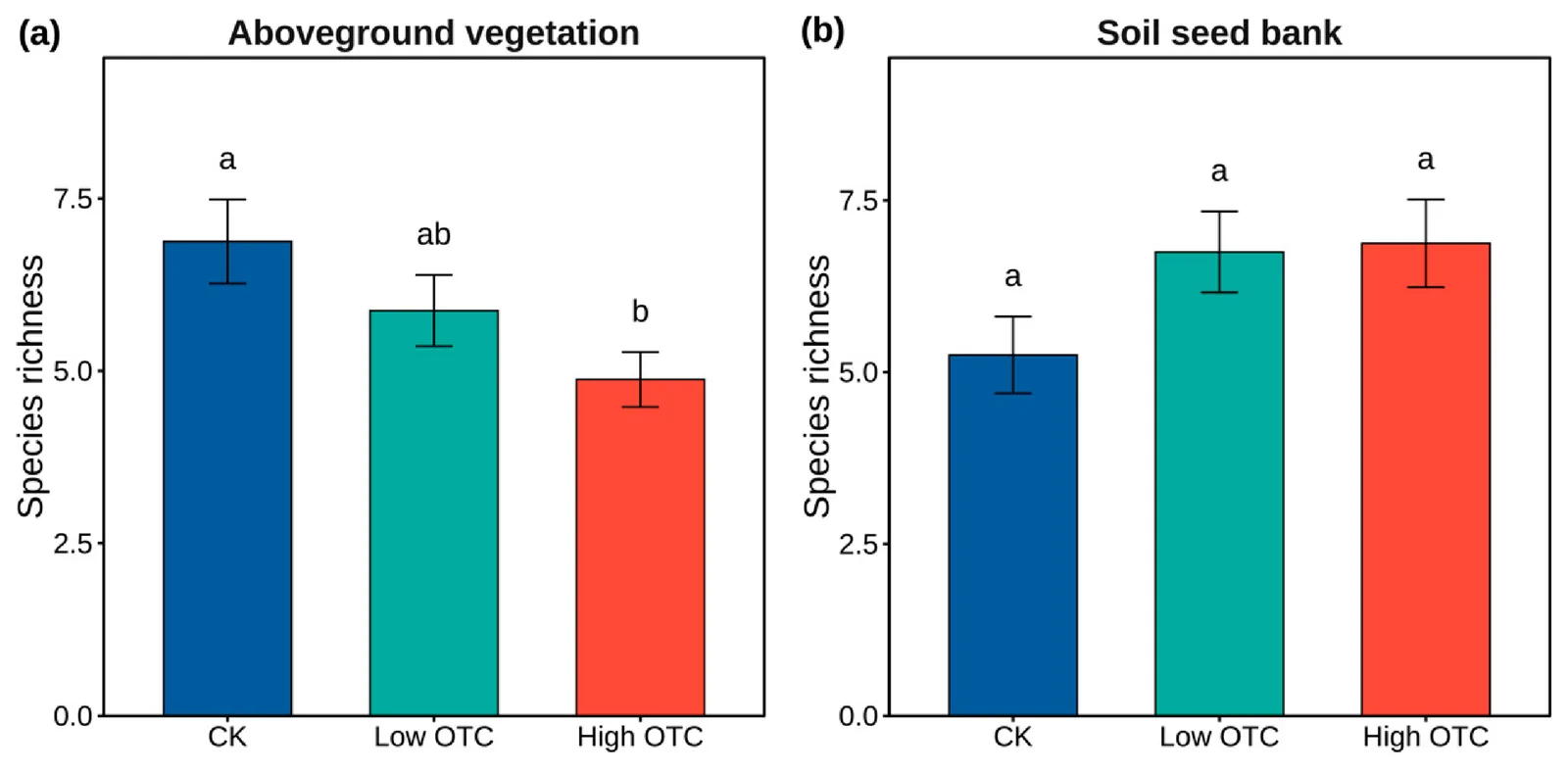
Effects of short-term experimental warming on species richness of aboveground vegetation (**a**) and the soil seed bank (**b**). Note: (**a**) Aboveground vegetation; (**b**) soil seed bank. High OTC and Low OTC represent the high- and low-intensity warming treatments, respectively, and CK represents the control shared by both warming treatments. Data are presented as the mean ± standard error (SE) of eight independent plots per treatment (n = 8). Different lowercase letters indicate significant differences among treatments based on one-way ANOVA followed by Tukey’s honestly significant difference (HSD) test (*p* < 0.05), whereas the same lowercase letters indicate no significant difference.

Species richness of aboveground vegetation (Figure 5a) and the soil seed bank (Figure 5b) exhibited contrasting responses to short-term experimental warming. With increasing warming intensity, species richness of aboveground vegetation gradually declined, whereas that of the soil seed bank showed an increasing trend. Compared with the CK treatment, the low OTC and high OTC treatments reduced species richness of aboveground vegetation by 14.53% and 29.07%, respectively, with the reduction being statistically significant under the high OTC treatment (*p* < 0.05). In contrast, species richness of the soil seed bank increased to 1.29 and 1.31 times that of the CK treatment under the low OTC and high OTC treatments, respectively.

**Figure 6:**
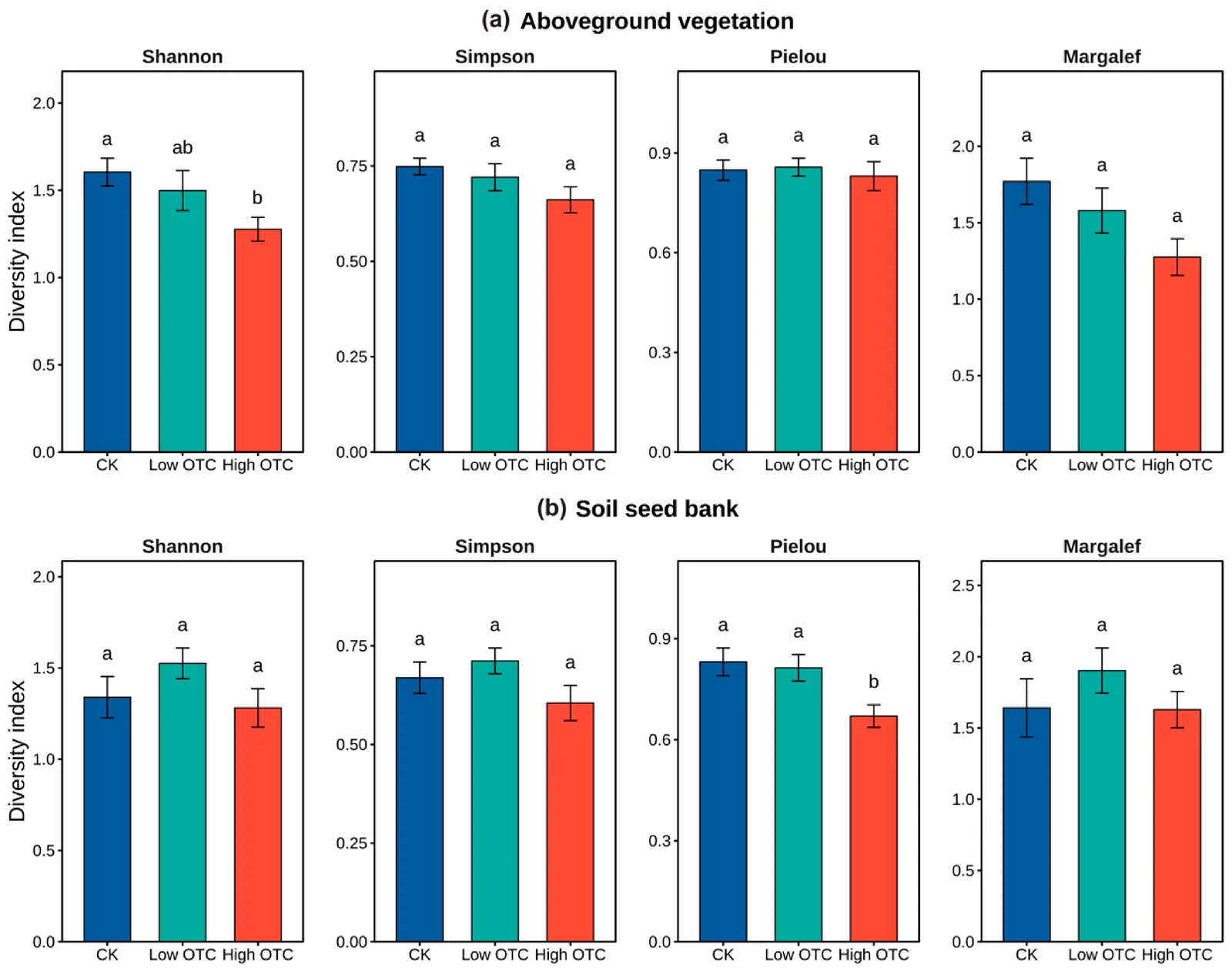
Effects of two years of experimental warming on the alpha diversity of aboveground vegetation (**a**) and the soil seed bank (**b**). Note: (**a**) Aboveground vegetation; (**b**) soil seed bank. High OTC and Low OTC represent the high- and low-intensity warming treatments, respectively, and CK represents the control shared by both warming treatments. Data are presented as the mean ± standard error (SE) (n = 8 independent plots per treatment). Different lowercase letters indicate significant differences among treatments based on one-way analysis of variance (ANOVA) followed by Tukey’s honestly significant difference (HSD) test (*p* < 0.05), whereas the same lowercase letters indicate no significant difference.

Further analysis revealed that the alpha-diversity indices of aboveground vegetation (Figure 6a) and the soil seed bank (Figure 6b) exhibited contrasting responses to two years of experimental warming. With increasing warming intensity, the Shannon diversity index of aboveground vegetation decreased by 5.03% and 22.64% under the low OTC and high OTC treatments, respectively, whereas the Pielou evenness index of the soil seed bank decreased by 2.41% and 19.28%, respectively. Both indices were significantly reduced under the high OTC treatment (*p* < 0.05). In addition, the Gini–Simpson and Margalef indices of aboveground vegetation showed decreasing trends with increasing warming intensity, whereas those of the soil seed bank remained relatively stable. However, neither index differed significantly among warming treatments (*p* > 0.05; Figure 6).

### 2.4. Effects of Warming on Beta Diversity of Aboveground Vegetation and the Soil Seed Bank

**Figure 7:**
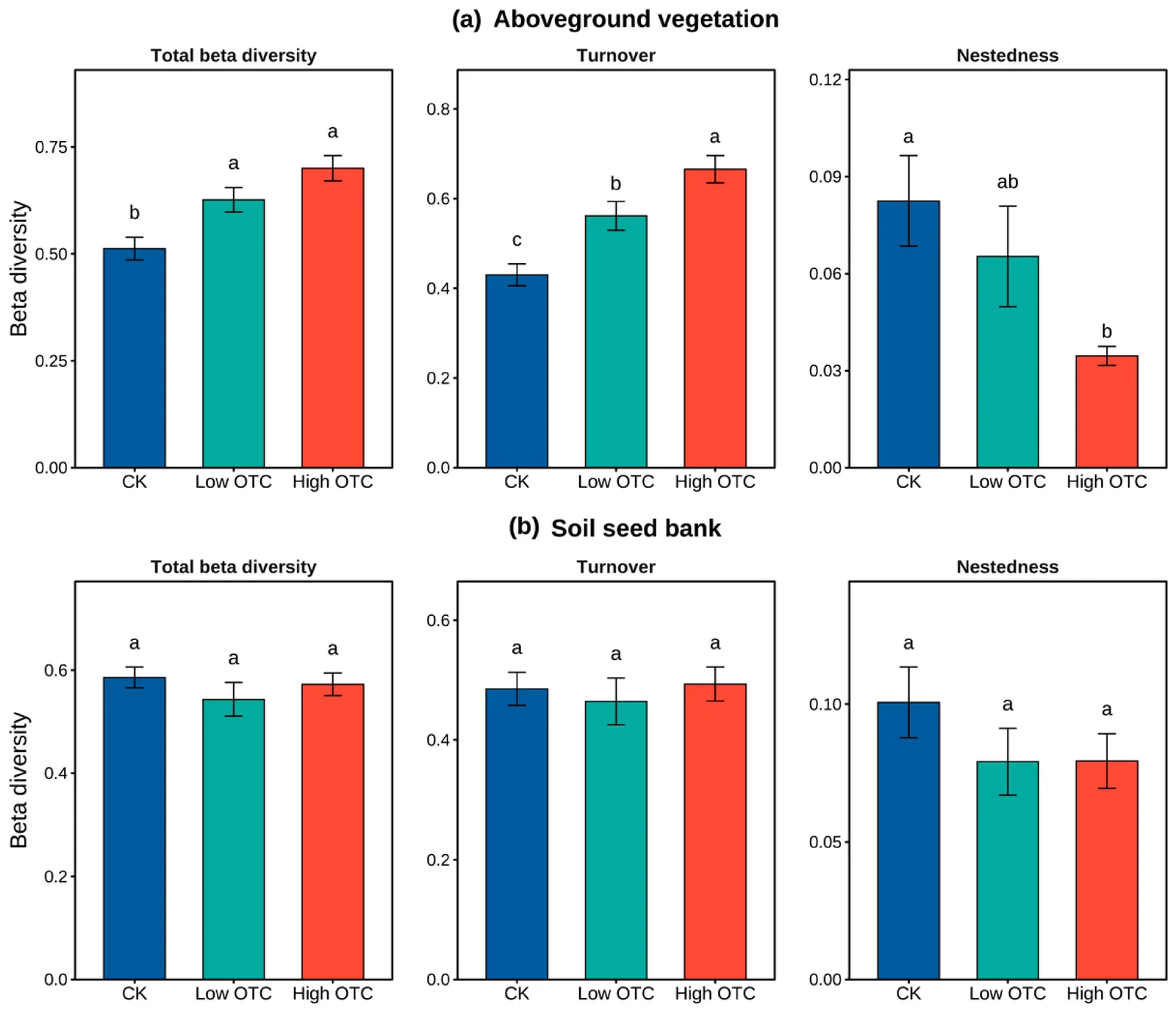
Effects of short-term experimental warming on the beta diversity of aboveground vegetation (**a**) and the soil seed bank (**b**). Note: (**a**) Aboveground vegetation; (**b**) soil seed bank. Values are presented as the mean ± standard error (SE) (n = 8 independent plots per treatment). Different lowercase letters indicate significant differences among treatments based on one-way analysis of variance (ANOVA) followed by Tukey’s honestly significant difference (HSD) test (*p* < 0.05), whereas the same lowercase letters indicate no significant difference.

Aboveground vegetation and the soil seed bank exhibited contrasting responses in beta diversity to short-term experimental warming (Figure 7). Compared with their respective controls, both the high OTC and low OTC treatments increased the beta diversity of aboveground vegetation by 37% and 24%, respectively (both *p* < 0.05), indicating that experimental warming enhanced differences in community composition among plots and promoted community heterogeneity. This increase in beta diversity was primarily driven by species turnover (Figure 7a). In contrast, the soil seed bank responded weakly to experimental warming (Figure 7b). With increasing warming intensity, total beta diversity decreased by 8% and 3%, respectively, indicating a trend toward community homogenization.

**Figure 8:**
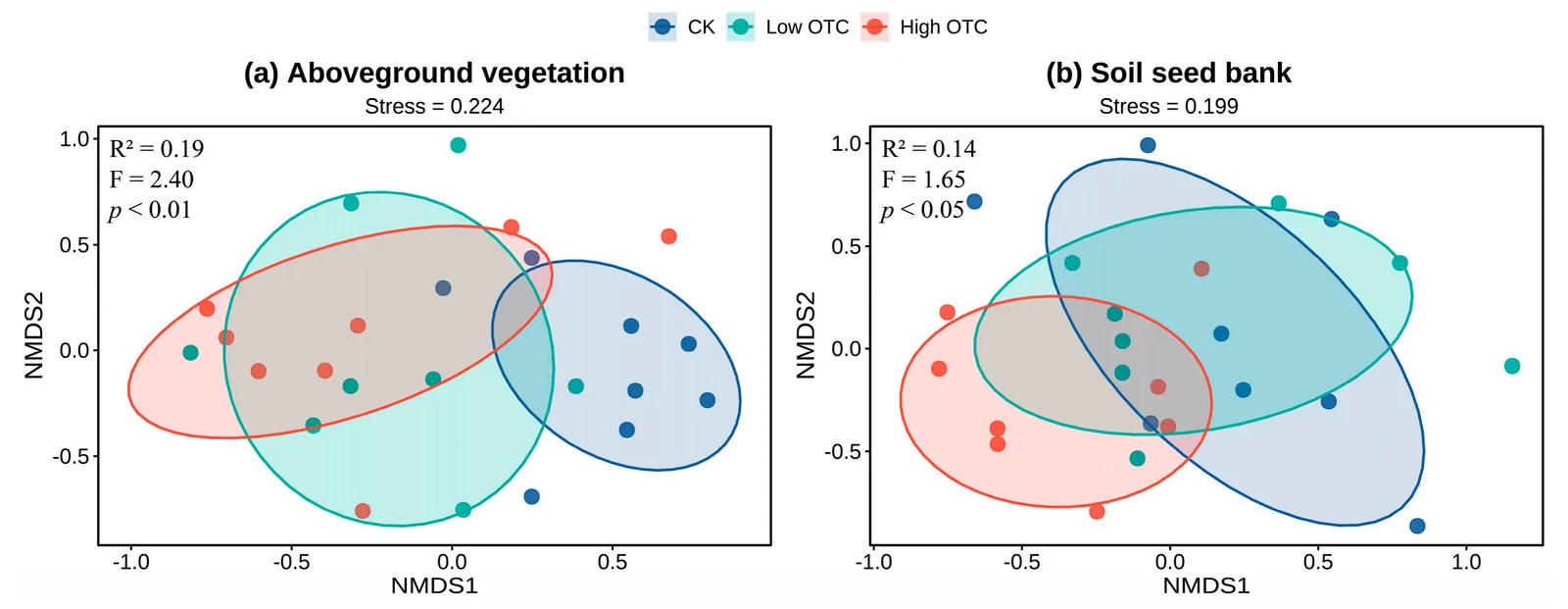
NMDS ordination showing the effects of short-term experimental warming on community composition of the aboveground vegetation (**a**) and the soil seed bank (**b**). Note: (**a**) NMDS ordination of the aboveground vegetation (Stress = 0.224; PERMANOVA: R^2^ = 0.19, F = 2.40, *p* < 0.01); (**b**) NMDS ordination of the soil seed bank (Stress = 0.199; PERMANOVA: R^2^ = 0.14, F = 1.65, *p* < 0.05).

To further evaluate the effects of experimental warming on community composition, non-metric multidimensional scaling (NMDS) and permutational multivariate analysis of variance (PERMANOVA) were performed (Figure 8). The NMDS ordination showed that samples of aboveground vegetation were more dispersed among treatments, whereas those of the soil seed bank exhibited greater overlap in ordination space. PERMANOVA indicated that experimental warming significantly affected the community composition of both aboveground vegetation (R^2^ = 0.19, F = 2.40, *p* < 0.01) and the soil seed bank (R^2^ = 0.14, F = 1.65, *p* < 0.05).

## 3. Discussion

### 3.1. Aboveground Vegetation Responds Rapidly to Short-Term Experimental Warming

Our results demonstrate that aboveground vegetation communities in alpine meadows respond rapidly during the early stage of experimental warming. Moreover, the effects on aboveground biomass became more pronounced with increasing warming intensity. The declines in species richness and the Shannon diversity index indicate that, although short-term warming enhanced community productivity, it did so at the expense of species diversity. This finding is consistent with previous studies showing that warming generally promotes aboveground biomass accumulation [11], while reductions in species richness have also been reported in some ecosystems under experimental warming [12]. The increase in aboveground biomass under experimental warming may be attributed to the long-term low-temperature limitation experienced by alpine meadows. Elevated temperatures may alleviate low-temperature limitation and promote plant growth in alpine meadows [10]. However, the effects of warming on ecosystem productivity are strongly influenced by water availability, and warming may even reduce productivity under water-limited conditions [10,27]. However, while warming stimulates rapid plant growth, it also intensifies interspecific competition, particularly by enhancing the ability of taller dominant species to capture light and occupy space [28]. Consequently, shorter or less competitive species are suppressed and are less able to complete normal growth and reproduction, ultimately resulting in reductions in community species richness and density. This interpretation is consistent with the findings of Klein et al. and Franklin et al. [28,29]. Meanwhile, the decline in the Shannon diversity index further indicates that warming reduced community evenness and compositional complexity, allowing dominant species to become increasingly dominant and the community to become progressively more dominance driven [30].

Notably, the decline in alpha diversity of the aboveground vegetation was accompanied by a gradual increase in beta diversity with increasing warming intensity, and this pattern was more pronounced under the high OTC treatment. These results indicate that short-term experimental warming not only reduced species diversity within communities but also increased community dissimilarity among treatments [31]. Consistent with the observed changes in beta diversity, the NMDS ordination and PERMANOVA analyses demonstrated that experimental warming significantly altered the community composition of the aboveground vegetation, indicating that short-term warming had already induced community reassembly. Furthermore, beta diversity partitioning showed that community differences were primarily driven by species turnover rather than nestedness [32], suggesting that warming-induced community changes resulted not simply from the random loss of species, but from species replacement among communities [33]. As temperature increased, species with greater thermal tolerance or stronger competitive ability gradually became dominant, whereas species with lower stress tolerance or weaker competitive ability gradually disappeared from the community. Consequently, changes in community composition were primarily driven by species replacement, rather than by simple species loss [28]. With increasing warming intensity, species replacement became progressively stronger, further enlarging differences in community composition among treatments and ultimately leading to an increase in beta diversity [34,35]. Therefore, our findings indicate that short-term experimental warming not only reduced the alpha diversity of aboveground vegetation but also promoted community reassembly through species replacement, ultimately increasing beta diversity. These results suggest that warming can simultaneously reshape the diversity pattern of aboveground vegetation in alpine meadows at both the within-community and among-community scales.

### 3.2. The Soil Seed Bank Exhibits Greater Stability Under Short-Term Experimental Warming

The soil seed bank, as an important source of propagules for plant community regeneration and restoration, was relatively less affected by short-term experimental warming. In the present study, experimental warming significantly increased soil seed bank density and promoted an increasing trend in species richness. Although beta diversity remained stable, PERMANOVA indicated that community composition responded significantly to warming (*p* < 0.05). However, the relatively low explanatory power (R^2^ = 0.14) together with the substantial overlap among treatments in the NMDS ordination suggests that the magnitude of this compositional change was relatively small. This result is consistent with the findings of Hoyle et al. [16], who reported that short-term warming primarily affected seed germination while causing little change in soil seed bank composition. However, Hoyle et al. [16] also reported that soil warming reduced overall germination despite increasing species richness, indicating that warming may influence different components of the germinable soil seed bank in different ways. Therefore, the increase in emerged seedlings observed in the present study should not be interpreted solely as enhanced germination but may also reflect changes in dormancy status or other unmeasured processes. These findings indicate that short-term experimental warming has not yet induced substantial community reassembly within the soil seed bank. This interpretation is supported by the NMDS ordination and PERMANOVA results, which together indicate that although warming significantly affected soil seed bank community composition, the magnitude of compositional change was considerably weaker than that observed for the aboveground vegetation [36,37].

The increase in the density of the germinable soil seed bank may reflect changes in seed dormancy status, overwinter survival, or germination potential induced by field warming. Because seed production, seed rain, dormancy status, and seed viability were not directly measured, these mechanisms remain speculative. Jeffrey L. Walck et al. [6] demonstrated that temperature is a key environmental factor regulating seed dormancy release and germination, and that moderate warming can promote dormancy release, thereby increasing seed germination capacity and the number of germinable seeds. In addition, experimental warming altered the soil microenvironment by increasing soil temperature, which may have influenced seed dormancy status or germination potential. However, because seed dormancy and overwinter survival were not directly measured in the present study, these mechanisms remain speculative. Previous studies have shown that alpine species differ in their germination strategies, suggesting that warming may influence different components of the germinable soil seed bank [38]. Therefore, we suggest that the increased density of the soil seed bank observed in this study more likely reflects an enhancement of the germination potential of existing seeds rather than the arrival of new species or the disappearance of existing ones.

Although experimental warming promoted seed germination, this effect mainly occurred in seeds already present in the soil and did not result in the establishment of new species or the loss of existing species. Consequently, no significant changes in beta diversity were observed. Walck et al. [6] reported that short-term warming primarily affects the release of seed dormancy and germination capacity rather than the species composition of the soil seed bank, which is consistent with our findings. Because the warming period was relatively short and soil possesses considerable buffering capacity, short-term warming was insufficient to alter soil seed bank diversity, further highlighting the stability of the soil seed bank.

### 3.3. Mechanisms Underlying the Differential Responses of Aboveground Vegetation and the Soil Seed Bank

Our results demonstrate that aboveground vegetation and the soil seed bank in alpine meadows exhibited contrasting responses to short-term experimental warming. Aboveground vegetation showed increased biomass, reduced alpha diversity, and increased beta diversity, whereas the soil seed bank exhibited only an increase in seed density while maintaining stable beta diversity. These contrasting responses indicate that aboveground vegetation is more environmentally sensitive to short-term warming than the soil seed bank, whereas the soil seed bank possesses greater stability and buffering capacity [14].

The contrasting responses between aboveground vegetation and the soil seed bank mainly arise from differences in their ecological functions and response timescales. As the component of the ecosystem directly exposed to the external environment, aboveground vegetation can rapidly adjust its growth, resource acquisition, and interspecific competition in response to temperature changes [10]. Short-term experimental warming altered aboveground community diversity, resulting in reduced alpha diversity and increased turnover-dominated dissimilarity among communities. These patterns indicate that warming influenced community composition, although the underlying ecological mechanisms were not directly evaluated in the present study. In contrast, the soil seed bank represents the potential component of plant communities and exhibits a pronounced historical legacy in its species composition [5]. Short-term warming primarily promoted dormancy release and germination of existing seeds [6,16], thereby increasing seed density without introducing new species or eliminating existing ones. As a result, both alpha diversity and beta diversity of the soil seed bank remained relatively stable.

In addition to differences in ecological function, aboveground vegetation and the soil seed bank also differ markedly in their response timescales [5]. Aboveground vegetation can respond rapidly to environmental change within a single growing season, whereas the response of the soil seed bank to warming requires a series of processes, including seed production, dispersal, incorporation into the soil, and subsequent survival before becoming fully expressed [6]. Consequently, although short-term experimental warming had already altered the diversity of aboveground vegetation, these changes had not yet accumulated sufficiently within the soil seed bank, resulting in contrasting responses in both alpha and beta diversity between the two community components. This asynchronous response suggests that detectable changes in the germinable soil seed bank lag behind those observed in aboveground vegetation during the early stage of experimental warming. Because the present study did not directly evaluate seed production, recruitment, or long-term vegetation recovery, the ecological consequences of this asynchronous response require further investigation [39].

However, several limitations should be acknowledged. First, passive open-top chambers (OTCs) inevitably modify multiple aspects of the microenvironment in addition to increasing temperature, including soil moisture, wind conditions, radiation, precipitation interception, and humidity. Because no sham chamber treatment was included in the present study, the observed ecological responses should be interpreted as the combined effects of OTC-based experimental warming rather than the effects of temperature alone. Second, the field experiment lasted only two growing seasons, whereas the formation and renewal of soil seed banks generally require much longer periods [5]. Therefore, the patterns observed here are likely to represent only the earliest stage of community reassembly following warming. Third, our analyses focused on community-level responses and did not directly quantify processes such as seed production, seed input, germination, or seed persistence [31]. Consequently, the pathways through which changes in aboveground vegetation are transmitted to the soil seed bank remain unresolved. Fourth, aboveground vegetation was surveyed only once during the peak growing season. Because experimental warming may alter plant phenology and senescence, species detectability may differ among treatments. Therefore, the absence of a species in a single survey should not be interpreted as evidence of local species loss, and repeated surveys throughout the growing season would provide a more comprehensive assessment of vegetation responses to warming. Future studies combining long-term field warming experiments with direct measurements of seed dynamics and repeated vegetation surveys will help determine whether the differential responses observed here ultimately alter aboveground and belowground diversity and thereby influence future community regeneration and successional trajectories.

## 4. Materials and Methods

### 4.1. Study Site

The study was conducted in an alpine meadow on the Qinghai–Tibetan Plateau, China (Figure 1a; 102°46′54.40″ E, 37°11′45.36″ N) [40]. The study area is situated at an elevation of 2862 m above sea level and is characterized by flat and open terrain, a cold and humid climate, thin air, and strong solar radiation. The mean annual air temperature is −0.1 °C, with January being the coldest month (mean temperature: −18.3 °C) and July the warmest month (mean temperature: 12.7 °C). The mean annual precipitation is 416 mm, with most rainfall occurring from July to September, while the mean annual evaporation reaches 1592 mm. The region experiences only two distinct seasons, namely a cold season and a warm season. The vegetation is a typical alpine meadow dominated by *Elymus nutans*, *Carex alatauensis*, and *Poa pratensis* L.

### 4.2. Experimental Design

**Figure 9:**
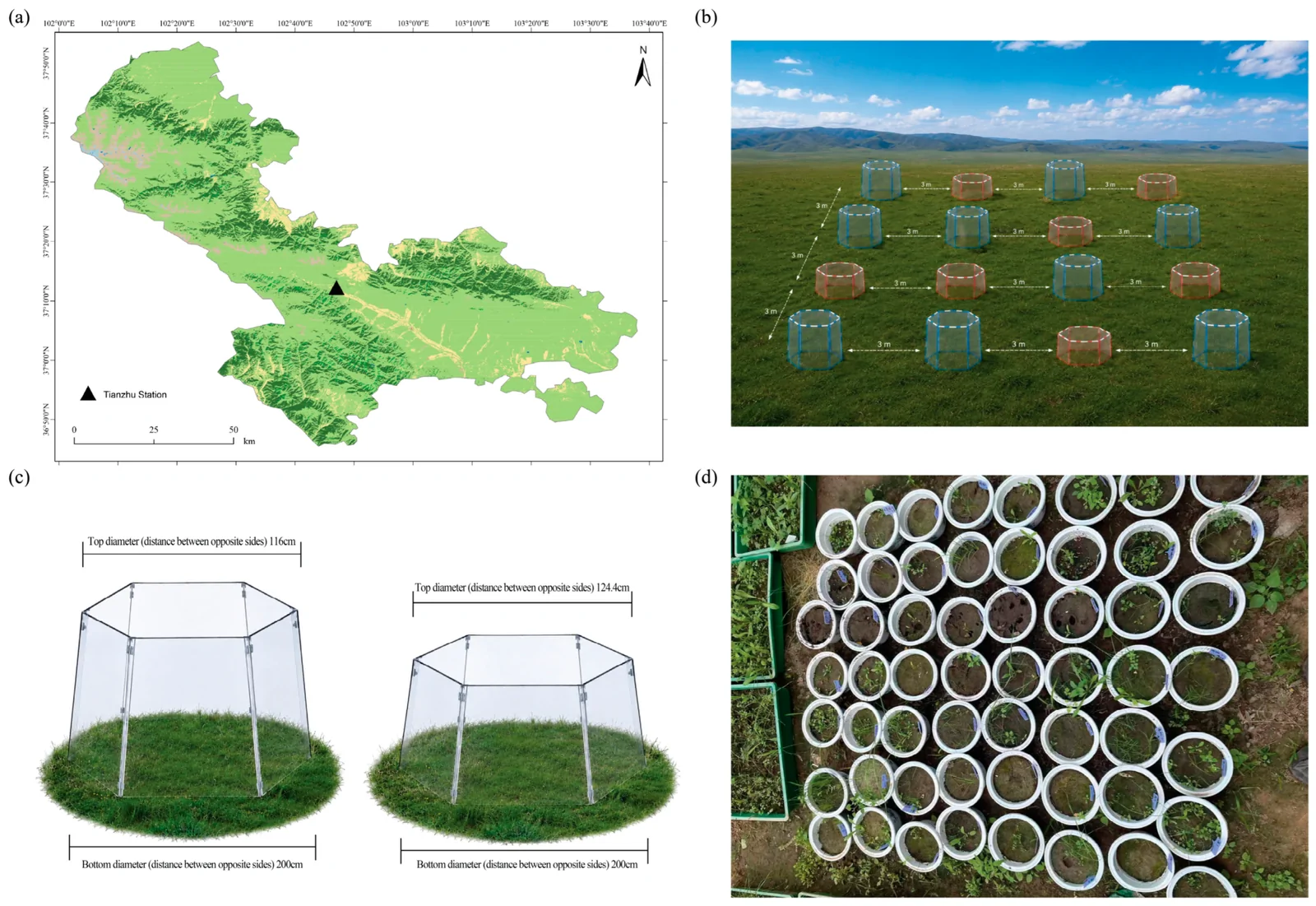
Location of the study area, field experimental layout, open-top chamber (OTC) design, and schematic illustration of the soil seed bank seedling emergence experiment. Note: (**a**) Geographic location of the Tianzhu Alpine Grassland Experimental Station; (**b**) field layout of the experimental plots showing the spatial arrangement of the sixteen permanent plots, treatment allocation, and the approximately 3 m spacing between adjacent plots; (**c**) structural dimensions of the high and low open-top chambers (OTCs); (**d**) schematic illustration of the soil seed bank seedling emergence experiment.

In April 2024, a simulated warming experiment was established at the Tianzhu Alpine Grassland Experimental Station of Gansu Agricultural University (an alpine meadow ecosystem; Figure 9a). The warming experiment was conducted over two consecutive growing seasons (2024 and 2025). A flat and open grassland without surrounding obstructions, such as walls or buildings, was selected for the experiment. Experimental warming was implemented using open-top chambers (OTCs) of two different heights [41]. The OTCs were constructed from transparent hexagonal acrylic panels with a light transmittance exceeding 90% [41,42]. Each chamber had a basal side length of 100 cm, a top opening side length of 116 cm, and was constructed from 5-mm-thick acrylic panels. The chambers remained installed continuously throughout the winter and the subsequent growing seasons. Two warming treatments were established, with both OTCs having a basal side length of 100 cm but differing in height: 70 cm for the high OTC treatment and 62 cm for the low OTC treatment. The experiment consisted of sixteen permanent plots arranged in a fixed 4 × 4 layout (Figure 9b,c). High OTC, Low OTC, and CK plots were arranged in a fixed alternating pattern with an inter-plot distance of approximately 3 m. Each warming treatment included eight independent replicates, and a single CK plot served as the control for both warming treatments. The experimental layout remained unchanged throughout the study and was not established as a randomized block design. Soil temperature (2 cm below the soil surface) and air temperature (20 cm above the ground surface) in both the warming and control plots were monitored from November 2024 to March 2025 using a high-precision Temp & Moisture Sensor (TMS, Prague, Czech Republic) [43]. Because the objective of this study was to evaluate the ecological responses of aboveground vegetation and the soil seed bank to experimental warming, temperature was used as the primary indicator of the warming treatment in the present analyses. The temperature records presented in Figure 1 correspond to this monitoring period.

### 4.3. Research Methods

#### 4.3.1. Soil Seed Bank Sampling

In this study, the seedling emergence method was used to estimate the germinable soil seed bank, rather than the total viable soil seed bank. Soil seed bank samples were collected from the high OTC, low OTC, and CK plots in late April 2025, before vegetation green-up, after approximately one year of experimental warming. To minimize disturbance to the long-term experimental plots and reduce edge effects, a random sampling point was established at the center of each plot. Soil samples were collected from the 0–10 cm soil layer using a square sampling frame with a side length of 16.8 cm. Each treatment (high OTC, low OTC, and CK) included eight replicates. A single CK treatment served as the control for both warming treatments, resulting in a total of 24 soil samples. After collection, all soil samples were immediately transported to the greenhouse at Gansu Agricultural University for the seedling emergence experiment. All soil samples were incubated under identical greenhouse conditions to provide uniform germination conditions rather than to simulate the natural alpine meadow environment. This approach minimized environmental variation during germination and ensured that differences in seedling emergence primarily reflected differences in the viable soil seed bank among warming treatments.

#### 4.3.2. Seedling Emergence Experiment

Because the seeds of many alpine meadow species are small and morphologically similar, making direct separation and identification difficult, the seedling emergence method was used to determine the composition of the soil seed bank [44]. After sampling, rhizomes and other vegetative propagules were manually removed from the soil samples. The soil from each sample was then evenly spread in plastic pots with an inner diameter of 21 cm. Each field soil sample was incubated in a single plastic pot, and no sample was subdivided among multiple pots. Each pot was pre-filled with a 5 cm layer of sterilized nutrient soil that had been oven-treated at 120 °C for 24 h to eliminate potential contamination from external seeds (Figure 9c). In addition, eight blank control pots containing only sterilized nutrient soil were established. No seedling emergence was observed in any of the blank control pots throughout the 120-day incubation period, confirming that the sterilized nutrient soil did not introduce detectable seed contamination into the experiment.

During the emergence experiment, the pots were watered regularly, and seedling emergence was continuously monitored. The emergence time, species identity, and number of seedlings were recorded. Seedlings that could be accurately identified were removed immediately after identification to avoid repeated counting. To promote the germination of ungerminated seeds remaining in the soil, the soil surface was gently disturbed once a week. After 120 days of incubation, a 500 mg L^−1^ gibberellic acid (GA_3_) solution was evenly applied to the soil surface of each pot until the soil was thoroughly moistened to promote the germination of dormant seeds. The experiment was terminated after no new seedlings had emerged for four consecutive weeks.

Seedlings were identified as soon as diagnostic morphological characteristics became visible. Individuals that could not be reliably identified at an early stage were retained until additional vegetative characteristics had developed. Species identification was based on the Flora of China and the Flora of Gansu, together with comparisons with mature plants collected from the study site. Taxonomic nomenclature was standardized according to the Plants of the World Online (POWO) database. Voucher specimens were not deposited because all identified species are common components of the study site and were identified based on vegetative morphological characteristics during the emergence experiment.

#### 4.3.3. Aboveground Vegetation Survey

The aboveground vegetation survey and biomass measurements were conducted in late July 2025 during the peak growing season. Because soil seed bank samples had previously been collected from the center of each plot, a separate 20 cm × 20 cm herbaceous quadrat was randomly established within the remaining undisturbed area of each plot for vegetation measurements, thereby avoiding any disturbance caused by soil-core sampling while minimizing potential edge effects associated with the OTCs. Each treatment (high OTC, low OTC, and CK) consisted of eight replicates, resulting in a total of 24 quadrats. All vegetation surveys were conducted by the same observer following a standardized field protocol. All plant species within each quadrat were recorded to determine species richness, plant density, and vegetation cover. Vegetation cover was visually estimated, whereas plant density was determined by directly counting visually distinguishable individual plants of each species within the quadrat. Following the vegetation survey, all aboveground plant material within each quadrat was clipped at ground level and collected. The samples were first heated at 105 °C for 30 min to terminate metabolic activity and then oven-dried at 65 °C to constant weight. The dry weight of each sample was subsequently determined and used as a measure of aboveground biomass. Biomass values were then converted to g m^−2^ by dividing the measured dry mass by the quadrat area (0.04 m^2^).

#### 4.3.4. Data Analysis

The species composition and seed density of the soil seed bank were evaluated based on the species identity and seed abundance obtained from the seedling emergence experiment. Soil seed bank density was standardized to the number of seeds per square meter according to the sampling area. The density of aboveground vegetation was standardized using the same approach. Species diversity of aboveground vegetation and the soil seed bank was quantified using the Shannon-Wiener diversity index [45], Gini–Simpson dominance index [46], Margalef richness index [47], and Pielou evenness index [48].

(1) $$\mathrm{Shannon}\text{-}\mathrm{Wiener}\;\mathrm{diversity}\;\mathrm{index}:\;H=-\overset{S}{\underset{i=1}{\sum }}P_{i}lnP_{i}$$

(2) $$\mathrm{Gini}\text{-}\mathrm{Simpson}\;\mathrm{dominance}\;\mathrm{index}:\;D=1-\overset{S}{\underset{i=1}{\sum }}P_{i}^{2}$$

(3) $$\mathrm{Margalef}\;\mathrm{richness}\;\mathrm{index}:R=\frac{S-1}{\ln\left(N\right)}$$

(4) $$\mathrm{Pielou}\;\mathrm{evenness}\;\mathrm{index}:\;E=\frac{H}{\ln\left(S\right)}$$

where N is the total number of individuals of all species in the soil seed bank or aboveground vegetation, Pi is the proportion of individuals belonging to species i relative to the total number of individuals, and S is the total number of species.

Total beta diversity among aboveground vegetation, among soil seed banks under different warming treatments, and between aboveground vegetation and the corresponding soil seed bank under the same warming treatment was calculated using the Sørensen dissimilarity index [49] via the beta.pair() function in the betapart package (version 1.6) in R [50]. Total beta diversity was further partitioned into turnover and nestedness components [32] using the same function. For each treatment group, pairwise Sørensen dissimilarities were calculated among all plots within that treatment; to avoid pseudoreplication, we then computed plot-level mean dissimilarities for each plot by averaging its dissimilarity to all other plots within the same treatment, yielding one beta-diversity value per plot (n = 8 per treatment). These plot-level mean values were used as the response variable in subsequent statistical analyses, with each plot treated as an independent replicate. When beta turnover exceeded beta nestedness, community dissimilarity was considered to be primarily driven by species replacement; conversely, when beta nestedness exceeded beta turnover, community dissimilarity was considered to be mainly attributable to species loss [50].

(5) $$\mathrm{Total}\;\mathrm{beta}\;\mathrm{diversity}:\beta_{SOR}=\frac{n+c}{2m+n+c}$$

(6) $$\mathrm{Beta}\;\mathrm{turnover}:\;\beta_{SIM}=\frac{\min\left(n,c\right)}{m+\min\left(n,c\right)}$$

(7) $$\mathrm{Beta}\;\mathrm{nestedness}:\beta_{SNM}=\frac{n+c}{2m+n+c}-\frac{\min\left(n,c\right)}{m+\min\left(n,c\right)}$$

where m is the number of species shared by the two communities, and n and c are the numbers of species unique to each of the two communities, respectively.

Non-metric multidimensional scaling (NMDS) based on Bray–Curtis dissimilarity was performed using the vegan package [51] in R (Version 4.3.3) to evaluate differences in community composition among samples.

All data processing, statistical analyses, and figure preparation were conducted in R (Version 4.3.3), primarily using the dplyr [52], tidyr [53], and ggplot2 [54] packages. Differences among treatments were tested using one-way analysis of variance (one-way ANOVA) followed by Tukey’s honestly significant difference (HSD) multiple comparison test. Differences were considered statistically significant at *p* < 0.05.

## 5. Conclusions

Experimental warming induced distinct response patterns in aboveground vegetation and the germinable soil seed bank of alpine meadows. Aboveground vegetation exhibited stronger responses to warming, as reflected by increased aboveground biomass, decreased alpha diversity, and increased beta diversity, indicating that short-term warming had already altered community structure through species replacement. In contrast, the germinable soil seed bank exhibited only an increase in seed density, whereas beta diversity remained relatively stable, suggesting that detectable changes in the germinable seed pool lagged behind those observed in aboveground vegetation during the early stage of experimental warming. Overall, this study demonstrates contrasting aboveground and belowground response patterns of alpine meadow plant communities to short-term experimental warming. However, because this study did not directly evaluate the compositional correspondence between aboveground vegetation and the germinable soil seed bank, nor did it assess long-term vegetation regeneration, the contribution of the germinable soil seed bank to future vegetation recovery remains uncertain and requires further long-term investigation. These findings improve our understanding of the early responses of alpine meadow plant communities to experimental warming and provide a basis for future studies on aboveground–belowground community dynamics under climate change.

## Data Availability

The data presented in this study are available on request from the corresponding author.

## Supplementary Materials

The following supporting information can be downloaded at: https://www.mdpi.com/article/10.3390/plants15162435/s1, Table S1: Species composition and density (individuals m^−2^) of aboveground vegetation; Table S2: Species composition and density (seeds m^−2^) of the soil seed bank. Table S3: Experimental timeline.
