# Supplementary material for: Contrasting α- and β-Diversity Responses of Aboveground Vegetation and Soil Seed Banks to Experimental Warming over Two Consecutive Growing Seasons
Source: Plants (Basel). 2026 Aug 11;15(16):2435. doi: 10.3390/plants15162435 (PMC13516621; doi:10.3390/plants15162435)
Supplement: Supplementary file 1 [file plants-15-02435-s001.zip › plants-4461474-supplementary.pdf]

Tables S1

Species composition and density (individuals m^{-2}) of aboveground vegetation

Family	Species	Life form	CK	Low OTC	High OTC
Amaranthaceae	*Chenopodium glaucum* L.	AH	125.00	75.00	150.00
Asteraceae	*Taraxacum mongolicum.*	PH	50.00	50.00	50.00
	Artemisia annua.	AH	50.00	—	150.00
	Aster altaicus Willd.	PH	100.00	500.00	200.00
Fabaceae	*Medicago ruthenica.*	PH	450.00	400.00	450.00
	Astragalus mongholicus Bunge.	PH	50.00	—	—
Poaceae	*Elymus nutans.*	PH	200.00	700.00	525.00
	Koeleria macrantha.	PH	1500.00	275.00	75.00
	Leymus secalinus (Georgi) Tzvelev.	PH	275.00	225.00	175.00
	Stipa capillata L.	PH	900.00	125.00	275.00
	Poa pratensis L.	PH	650.00	25.00	—
Rosaceae	*Potentilla bifurca* L.	PH	100.00	250.00	200.00
Cyperaceae	*Carex alatauensis.*	PH	—	125.00	75.00
	Carex tristachya Thunb.	PH	75.00	200.00	75.00
	Carex capillifolia (Decne.) S. R. Zhang.	PH	325.00	250.00	100.00
Ranunculaceae	*Thalictrum alpinum.*	PH	225.00	675.00	75.00
Orobanchaceae	*Pedicularis kansuensis Maxim.*	AH	—	—	50.00
Gentianaceae	*Gentiana macrophylla Pall.*	PH	50.00	50.00	25.00
Caryophyllaceae	*Silene sp.*	PH	25.00	—	25.00
	Silene conoidea.	AH	200.00	100.00	50.00
Iridaceae	*Iris lactea Pall.*	PH	—	75.00	—
Total			5350.00	4100.00	2725.00

Note: "—" indicates not detected or not recorded. AH = annual herb; PH = perennial herb.

Tables S2

Species composition and density (seeds m^{-2}) of the soil seed bank

Family	Species	Life form	CK	Low OTC	High OTC
Amaranthaceae	*Chenopodium glaucum* L.	AH	13.29	22.14	70.86
Asteraceae	*Taraxacum mongolicum.*	PH	22.14	4.43	8.86
	Aster alpinus L.	PH	4.43	8.86	8.86
	Artemisia annua.	AH	132.87	79.72	150.58
	Erigeron canadensis L.	AH	8.86	4.43	8.86
	Artemisia frigida Willd.	PH	8.86	8.86	8.86
Polygonaceae	*Bistorta vivipara.*	PH	—	4.43	—
Fabaceae	*Medicago ruthenica.*	PH	—	—	17.72
	Oxytropis ochrocephala Bunge.	PH	—	—	4.43
Poaceae	*Poa pratensis* L.	AH	124.01	181.58	442.89
	Elymus nutans.	PH	141.72	256.87	442.89
	Agropyron cristatum.	PH	8.86	—	—
	Elymus sibiricus L.	PH	—	4.43	—
Lamiaceae	*Elsholtzia densa.*	AH	4.43	—	—
	Dracocephalum heterophyllum.	PH	—	4.43	8.86
Rosaceae	*Potentilla anserina.*	PH	4.43	8.86	22.14
	Potentilla bifurca L.	PH	4.43	26.57	26.57
Papaveraceae	*Hypecoum leptocarpum.*	AH	13.29	—	93.01
Salicaceae	*Salix myrtillacea Andersson.*	S	17.72	17.72	22.14
Tamaricaceae	*Tamarix chinensis Lour.*	S	13.29	22.14	4.43
Boraginaceae	*Lappula myosotis.*	AH	—	8.86	—
Plantaginaceae	*Plantago asiatica.*	PH	—	8.86	—
Caryophyllaceae	*Stellaria umbellata Turcz.*	PH	—	4.43	13.29
Gentianaceae	*Gentiana macrophylla Pall.*	PH	—	4.43	17.72
Ranunculaceae	*Anemone sp.*	PH	—	—	4.43
Brassicaceae	*Lepidium apetalum Willd.*	AH	48.72	119.58	44.29
Primulaceae	*Glaux maritima* L.	PH	4.43	—	—
Portulacaceae	*Portulaca oleracea* L.	AH	—	4.43	—
Molluginaceae	*Trigastrotheca stricta (*L.*) Thulin.*	AH	—	17.72	—
Total			575.78	823.78	1421.69

Note: "—" indicates not detected or not recorded. AH = annual herb; PH = perennial herb.

Tables S3

Experimental timeline

**Time**	**Event**
April 2024	OTC installation
November 2024–March 2025	Microclimate monitoring
Late April 2025	Soil seed bank sampling
Late April 2025	Seedling emergence experiment initiated
Late July 2025	Aboveground vegetation survey
120 days after germination monitoring began	GA_3 application
End of the emergence experiment	Final census and experiment termination
